# Interactions between *Yersinia pestis* V-antigen (LcrV) and human Toll-like receptor 2 (TLR2) in a modelled protein complex and potential mechanistic insights

**DOI:** 10.1186/s12865-019-0329-5

**Published:** 2019-12-16

**Authors:** Tiandi Wei, Jing Gong, Guojing Qu, Mingyu Wang, Hai Xu

**Affiliations:** 1 State Key Laboratory of Microbial Technology, Microbial Technology Institute, Shandong University, Qingdao, China; 2 Taishan College, Shandong University, Qingdao, China; 3 School of Life Sciences, Shandong University, Qingdao, China

**Keywords:** *Yersinia pestis*, LcrV, V-antigen, Toll-like receptor, TLR2, Plague, Structure modelling, Immune response repression

## Abstract

**Background:**

*Yersinia pestis*, the etiological pathogen of plague, is capable of repressing the immune response of white blood cells to evade phagocytosis. The V-antigen (LcrV) was found to be involved in this process by binding to human Toll-like Receptor 2 (TLR2). The detailed mechanism behind this LcrV and TLR2 mediated immune response repression, however, is yet to be fully elucidated due to the lack of structural information.

**Results:**

In this work, with protein structure modelling, we were able to construct a structure model of the heterotetramer of *Y. pestis* LcrV and human TLR2. Molecular dynamics simulation suggests the stability of this structure in aquatic environment. The LcrV model has a dumbbell-like structure with two globule domains (G1 at N-terminus and G2 away from membrane) connected with a coiled-coil linker (CCL) domain. The two horseshoe-shape TLR2 subunits form a V-shape structure, are not in direct contact with each other, and are held together by the LcrV homodimer. In this structure model, both the G1 and CCL domains are involved in the formation of LcrV homodimer, while all three domains are involved in LcrV-TLR2 binding. A mechanistic model was proposed based on this heterotetrameric structure model: The LcrV homodimer separates the TLR2 subunits to inhibit the dimerization of TLR2 and subsequent signal transfer for immune response; while LcrV could also inhibit the formation of heterodimers of TLR2 with other TLRs, and leads to immune response repression.

**Conclusions:**

A heterotetrameric structure of *Y. pestis* LcrV and human TLR2 was modelled in this work. Analysis of this modelled structure showed its stability in aquatic environments and the role of LcrV domains and residues in protein-protein interaction. A mechanistic model for the role of LcrV in *Y. pestis* pathogenesis is raised based on this heterotetrameric structure model. This work provides a hypothesis of LcrV function, with which further experimental validation may elucidate the role of LcrV in human immune response repression.

## Background

*Yersinia pestis* is a deadly pathogen that caused three of the most catastrophic plagues in human history, including the notorious “Black Death” in Europe in Mid 1300’s, leading to the deaths of approximately 17 to 28 million people [1, 2]. Today, despite extreme precautions that were taken in order to prevent the outbreak of *Y. pestis*, cases of *Y. pestis* infection that frequently result in patient deaths were still reported now and then [3]. Infection of *Y. pestis* is commonly mediated by bacteria-containing aerosol inhalation or flea bite that transmits the bacterium from pathogen-carrying reservoir mammal hosts to human, leading to rapid progression of symptoms from fever to pneumonia, to hemoptysis, and eventually to patient deaths in 3–4 days [4, 5].

One striking feature of *Y. pestis* is its ability to evade phagocytosis and grow in white blood cells such as macrophages [6]. This was done by injection of *Yersinia* outer membrane proteins (Yops) to cells by Type III Secretion System (T3SS, also termed the injectisome) upon contact with target cells [7, 8]. The injected Yops subsequently repress phagocytosis and the immunity-related signal pathways [9]. Gene encoding these proteins reside on the virulence plasmid pYV (also termed pCD) that’s co-hosted by a series of pathogenic *Yersinia* species such as *Y. pestis*, *Yersinia pseudotuberculosis*, and *Yersinia enterocolitica* [9–11]. *Y. pseudotuberculosis* and *Y. enterocolitica* are enteric members of the *Yersinia* genus that are transmitted primarily by contaminated food and water. These two species do not cause plagues but rather leads to a variety of diseases such as enterocolitis [12]. The pYV plasmid also carries a *lcrV* gene that encodes a Low Calcium Response V (LcrV, also termed the V-antigen) protein. This protein has been considered important in the virulence of *Y. pestis*.

The role of LcrV in the pathogenesis of *Y. pestis* has been previously investigated in a variety of contradictory reports. LcrV was found secreted to the extracellular space to assist the entry of Yops to host cells [13, 14]. It was later found that LcrV leads to immune response repression by improving IL-10 expression and subsequently repressing inflammation factors TNF-α and IFN-γ in *Y. enterocolitica* [15, 16]. This response was found to be mediated by the binding of host Toll-like receptor 2 (TLR2) and LcrV at two independent binding sites (L32-L35 and D203-I206) [17–19]. Different signal transduction pathways were also proposed, suggesting that LcrV can repress TFN-α via a yet unknown IL-20 independent pathway [20]. However, in a report by Pouliot et al., controversy arose as the authors found *Y. pestis* TLR2 cannot be activated by LcrV and therefore is not able to mediate IL-10-dependent immune response by LcrV [21]. This finding was supported by a subsequent investigation showing *Y. pestis* LcrV cannot lead to significant IL-10 induction [22].

In order to further understand the role of LcrV in the pathogenesis of *Y. pestis* and the molecular mechanism by which LcrV represses immune response, structural information is needed for this protein, as well as for the interaction between this protein and its potential targets. The crystal structure of an entropy reduced mutant of *Y. pestis* LcrV was obtained at 2.2 Å [1]. However, this structure was mutated at K40-K42, was incomplete at loop regions, and was monomeric despite reports suggesting LcrV is a homodimer [23]. Later attempts were able to solve the LcrV structure at 1.65 Å [24]. This structure, however, is also incomplete for the lack of C-terminal loop structures. No investigations have been reported on the structure of the LcrV-TLR2 complex. This lack of structural knowledge prevents us from further elucidating the interaction of LcrV and TLR2, as well as further understanding the role of LcrV in *Y. pestis* pathogenesis.

In this work, aiming at providing further structural information on the LcrV-TLR2 complex, we attempted to apply bioinformatical methods to predict the interaction between *Y. pestis* LcrV and *H. sapiens* TLR2. A heterotetrameric model was constructed and evaluated by molecular dynamic simulations in an aquatic system. Based on this structural model, we are able to predict structural contacts between LcrV and TLR2, and identify key regions essential for LcrV function. A model on the mechanism by which LcrV regulates immune response is raised.

## Results

### Modelling and assessment of the LcrV-TLR2 complex structure

Two X-ray diffraction structures (PDB ID: 1R6F and 4JBU) were previously reported for mutants of *Y. pestis* LcrV. The 2.17 Å 1R6F structure mutated KDK_40–42_ to AAA, deleted Y_90_, lacked D_51_ to N_51_ and N_263_ to C_273_. The 1.65 Å 4JBU structure lacked N_263_ to P_279_. These flaws were fixed by performing homologous modelling of the G_28_-D_322_ fragment of *Y. pestis* LcrV (Uniprot accession P0C7U7, full length 326 AA) using these two reported structures as templates. A similar approach was done to obtain the modelled structure of TLR2 extracellular domain (Uniprot accession O60603) based on the previously reported *H. sapiens*-hagfish fusion TLR2 structure (PDB ID 2Z7X) and *Mus musculus* TLR2 structure (PDB ID 5D3I). The modelled LcrV and TLR2 structures were evaluated to confirm their quality (Additional file 1). The heterotetrameric LcrV-TLR2 complex structure model was subsequently constructed by consecutive modelling the LcrV dimeric structure, LcrV-TLR2 heterodimeric structure, and ultimately the LcrV-TLR2 heterotetrameric structure.

**Fig. 1:**
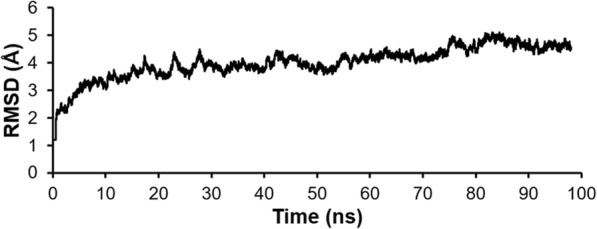
Stability of modelled LcrV-TLR2 complex structure in water environment

The stability of the LcrV-TLR2 heterotetrameric structure model was assessed by performing molecular dynamics analysis of the structure in water environments over a time frame of 100 ns and time interval of 10 ps (Fig. 1). The RMSD of the structure (in comparison with the modelled heterotetrameric structure) stabilized after 20 ns, reaching approximately 5 Å at the end of 100 ns. This assessment suggests that the LcrV-TLR2 heterotetrameric structure model in a water environment is stable, confirming the quality of the structure.

### Overall structure of the modelled LcrV-TLR2 complex

**Fig. 2:**
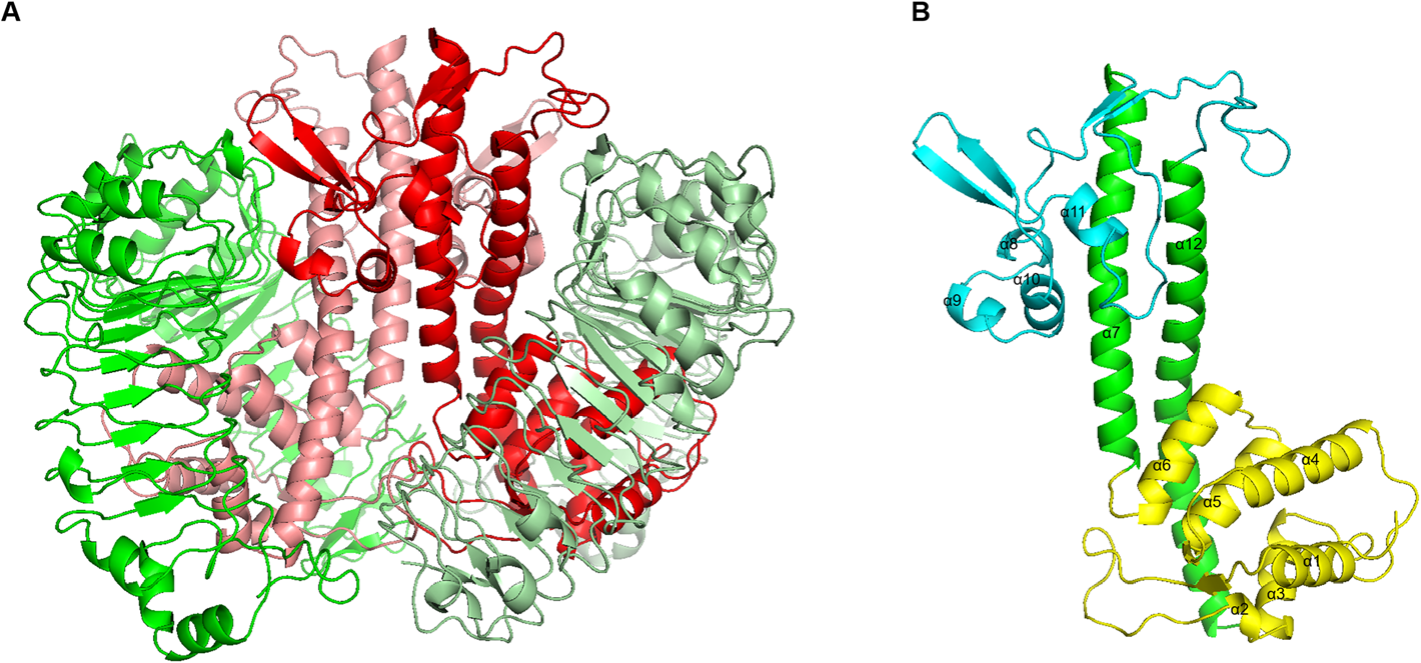
Modelled structures of *Y. pestis* LcrV and the LcrV-TLR2 heterotetramer. Panel (**a)**, LcrV-TLR2 complex structure model, shown in red colors are LcrV subunits, shown in green colors are TLR2 periplasmic fragments; Panel (**b**), LcrV monomer, yellow color indicates the N-terminal globule module (Domain G1), blue color indicates the C-terminal globule module (Domain G2), green color indicates the coiled-coil structure connecting the two globule modules (Domain CCL), α helices are indicated

The overall modelled structure of the LcrV-TLR2 complex is a heterotetramer formed by two *Y. pestis* LcrV subunits and two *H. sapiens* TLR2 subunits (Fig. 2a). The modelled LcrV monomer has a unique dumbbell-shape structure: two globule modules connected by a long coiled-coil structure formed by two long antiparallel α helices (Fig. 2b), in consistence with previously solved crystal structures of LcrV. The three modules are respectively termed Domain G1 (membrane-adjacent globule), CCL (coiled-coil linker), and G2 (loop-rich globule away from membrane). Domain G1 in modelled LcrV structure is formed by six α-helices, of which α1 and α2 are connected by a long loop. Domain G2 is a loop-rich globule module stabilized with two antiparallel β strand pairs and four short α helices. A β-hairpin structure connects α7 and α8, while a long loop connects α11 and α12 in the modelled structure.

In this modelled LcrV-TLR2 heterotetramer, two horseshoe-like TLR2 subunits form a V-shaped structure with a dihedral angle of approximately 70 degrees, with their openings facing towards the membrane. The two LcrV structures are sandwiched between the two TLR2 subunits in this model (Fig. 2a). The two TLR2 subunits have very few direct contacts in the model. Instead, they were held together by the two LcrV subunits, forming a LcrV-TLR2 heterotetrameric complex.

### Proposed basis for LcrV-TLR2 heterotetramer formation

**Fig. 3:**
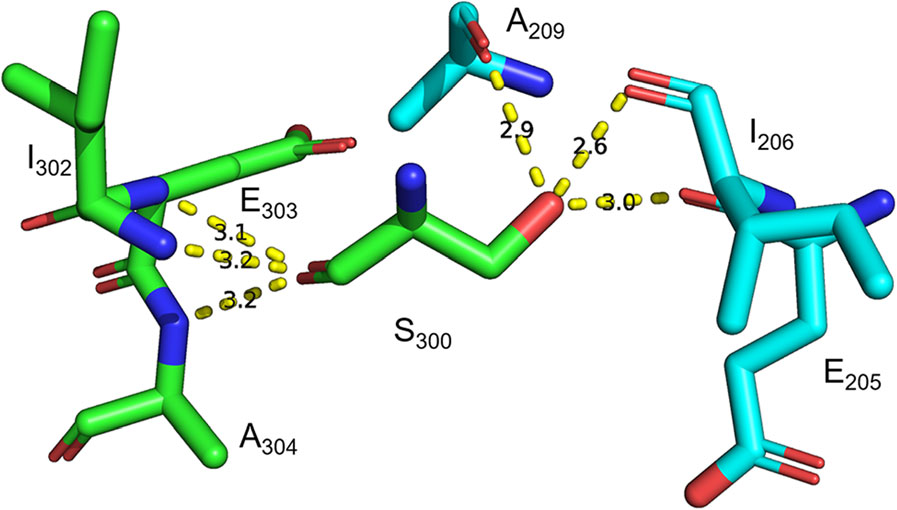
Hydrogen bonds formed by S_300_ in structure model. Dashed lines indicate potential hydrogen bonds. Numbers indicate bond length (in Å). Green and light blue color backbones indicate two different LcrV monomers. Blue color indicates nitrogen atoms. Red color indicates oxygen atoms

Analysis of the modelled LcrV-TLR2 heterotetramer leads to the proposal that the dimeric LcrV structure is formed via the contacts in primarily Domain G2 and CCL. The extended loop region between β1 and α2 in Domain G1 (YDP_50–52_ and EVFA_57–60_) could form contacts between the two monomers, potentially by π-π stacking between Y_50_. The two α7 in Domain CCL in each monomer form close contacts, and are potentially held together by hydrogen bonds between R_150_ and S_151_ (4.1 Å), as well as between R_154_ and E_155_ (3.0–4.1 Å). The α9 (GYTDEEIFKA_200–209_) of Domain G2 forms close contacts with α12 (SDITSRKNSAIEA_292–304_) of Domain CCL. This contact is formed via a hydrogen bond network: the hydroxyl group of Y_201_ (donor) forms a hydrogen bond with the side chain carboxamide of N_299_ (acceptor, 2.6–3.8 Å); while the hydroxyl group of S_300_ forms hydrogen bonds with the peptidyl carbonyl group (acceptor) of A_209_ (2.9 Å), I_206_ (2.6 Å), and E_205_ (3.0 Å) (Fig. 3). Interestingly, the peptidyl carbonyl group (donor) of S_300_ forms hydrogen bonds with the peptidyl amino group (acceptor) of I_302_ (3.2 Å), E_303_ (3.1 Å), and A_304_ (3.2), suggesting the key role of this residue in the formation of the hydrogen bond network for intact dimeric structure formation.

**Fig. 4:**
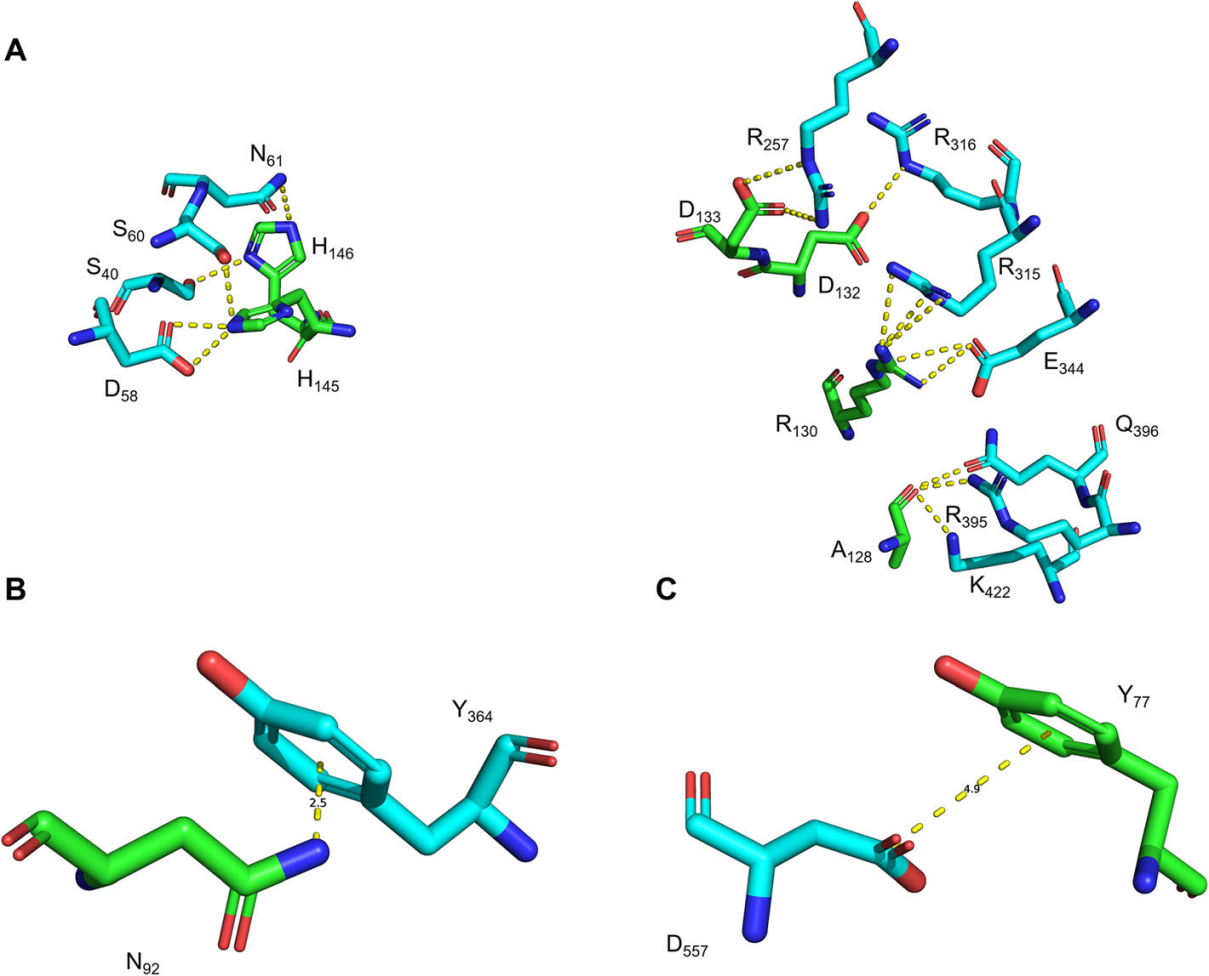
Proposed interactions between TLR2 and LcrV Domains G1/CCL on the same side. Panel (**a**): hydrogen bond network, dashed lines indicate potential hydrogen bonds; Panel (**b**), cation-π interaction between LcrV N_92_ and TLR2 Y_364_; Panel (**c**), π-π interaction between LcrV Y_77_ and TLR2 D_557_. Green and light blue color backbones respectively indicate TLR2 and LcrV. Blue color indicates nitrogen atoms. Red color indicates oxygen atoms. Numbers indicate bond length (in Å)

**Fig. 5:**
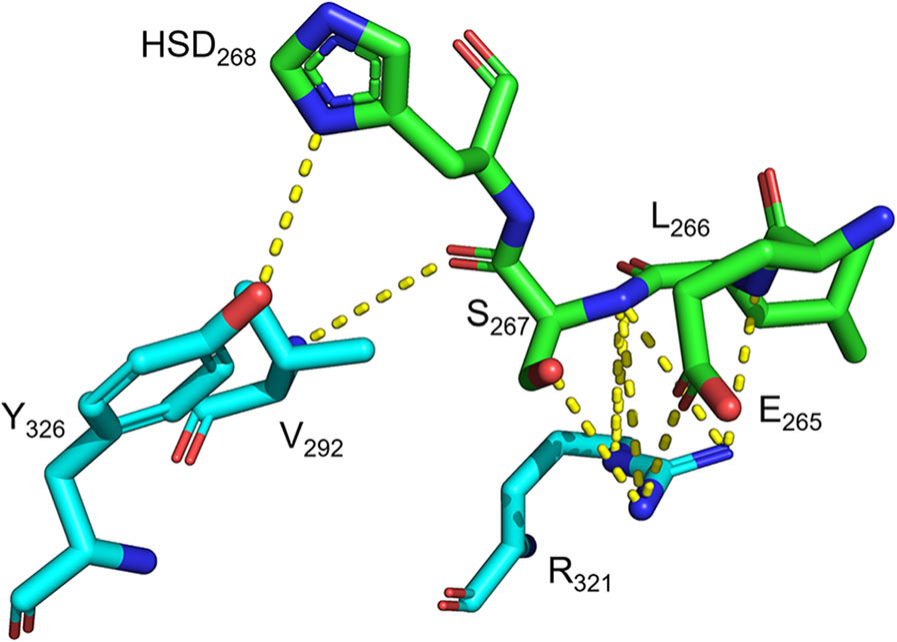
Proposed interactions between TLR2 and LcrV Domain G2 on the same side. Dashed lines indicate potential hydrogen bonds. Green and light blue color backbones respectively indicate TLR2 and LcrV. Blue color indicates nitrogen atoms. Red color indicates oxygen atoms

Further analysis of the heterotetrameric LcrV-TLR2 structure model suggests both LcrV subunits potentially form contacts with each TLR2 subunit. The LcrV subunit on the ‘same side’ shows extensive contacts with TLR2 in all three domains in the model. A total of 20 hydrogen bonds are formed between Domain G1 and TLR2 (Table 1). These hydrogen bonds form a network that fits Domain G1 in the hollow center of the horseshoe like TLR2 structure. In particular, two regions, namely ADRIDD_128–133_ and H_145_H_146_, are two hubs for hydrogen bond formation and may play key roles in the binding of LcrV and TLR2 (Fig. 4a). Two additional interactions are also involved in the binding of Domain G1 and TLR2: the cation-π interaction between LcrV N_92_ and TLR2 Y_364_ (Fig. 4b), as well as the π-π interaction between LcrV Y_77_ and TLR2 D_557_ (Fig. 4c). The LcrV Domain G2 loop region between β6 and α12, namely ELS_265–267_ and a histidine derivative at position 268, is another key location for the binding to TLR2 due to the hydrogen bond network between these residues and TLR2 (Fig. 5). In LcrV Domain CCL, α12 forms an extensive hydrogen bond network with TLR2, with 19 predicted hydrogen bonds formed (Table 2).

**Table 1:** Predicted hydrogen bonds between LcrV Domain G1 and TLR2 on the same side

LcrV residue	LcrV secondary structure	TLR2 residue	Bond length (Å)
N_43_	Linker between α1 and β1	E_103_	3.0
Q_93_	α4	Y_364_	4.0
N_96_	α4	R_340_	3.0
K_99_	α4	K_253_	3.5
R_100_	α4	R_315_	3.6
E_106_	α4	E_178_	3.0
Q_112_	Linker between β2 and α5	K_37_	2.6
A_128_	Loop between α5 and α6	R_395_	2.8
A_128_	Loop between α5 and α6	Q_396_	2.8
A_128_	Loop between α5 and α6	K_422_	2.8
R_130_	Loop between α5 and α6	R_315_	3.0
R_130_	Loop between α5 and α6	E_344_	3.5
D_132_	Loop between α5 and α6	R_316_	3.9
D_133_	α6	R_257_	3.3
K_137_	α6	Y_109_	4.2
H_145_	α6	S_39_	3.8
H_145_	α6	D_58_	2.7
H_145_	α6	S_60_	3.3
H_146_	Linker between α6 and α7	N_61_	3.2
H_146_	Linker between α6 and α7	S_40_	3.0

**Table 2:** Predicted hydrogen bonds between LcrV Domain CCL and TLR2 on the same side

LcrV residue	LcrV secondary structure	TLR2 residue	Bond length (Å)
D_294_	α12	H_318_ (two nitrogen atoms on side chain imidazole group)	3.0
			3.1
D_294_	α12	R_316_ (three side chain amino groups)	2.6
			2.8
			3.1
R_297_	α12	R_316_ (two side chain amino groups)	2.6
			3.0
R_297_	α12	D_286_	4.0
K_311_	α12	R_486_	3.7
R_318_	α12	G_532_ (peptidyl carbonyl group)	3.6
R_318_	α12	G_532_ (peptidyl amino group)	3.2
L_320_	α12	Q_574_	3.4
D_321_	α12	Q_574_	3.4
D_321_	α12	N_561_	3.5
D_321_	α12	Y_562_	3.9
D_321_	α12	L_563_	3.5
D_322_ (two side chain carbonyl groups)	α12	N_561_	3.1
			3.3
D_322_	α12	W_558_	3.8

**Fig. 6:**
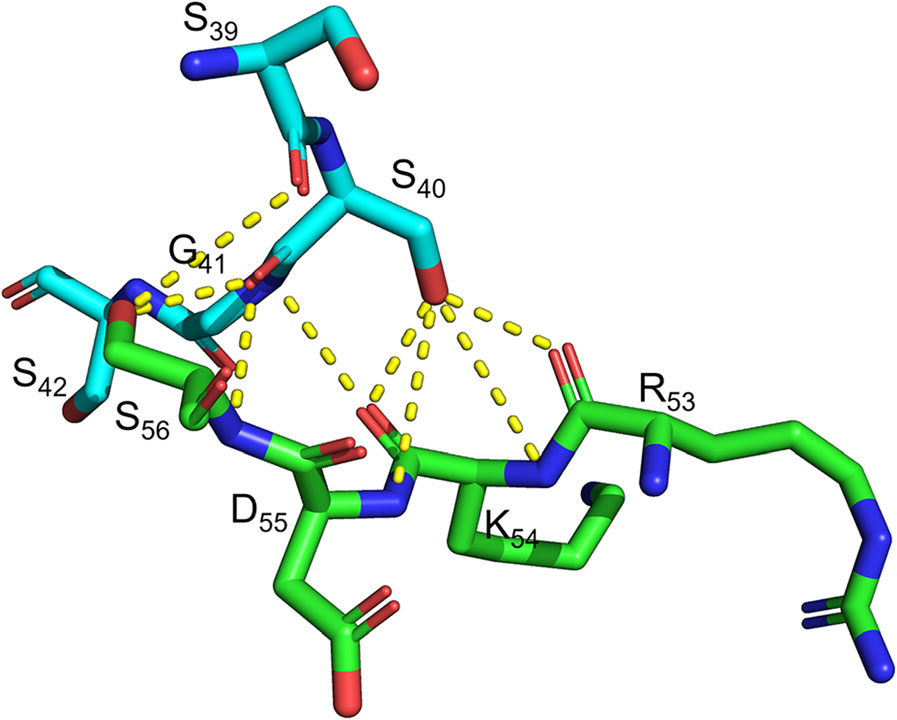
Proposed hydrogen bond network between TLR2 SSGS_39–42_ and LcrV RKDS_53–56_ on the opposite side. Dashed lines indicate potential hydrogen bonds. Green and light blue color backbones respectively indicate TLR2 and LcrV. Blue color indicates nitrogen atoms. Red color indicates oxygen atoms

The LcrV subunit on the ‘opposite side’ also forms close contacts with TLR2 subunit in the model, reinforcing the LcrV-TLR2 heterotetramer formation. Three regions are involved in the interaction between the ‘opposite side’ LcrV and TLR2: the loop region between β1 and α2 in Domain G1, the α8-α9 linker and the beginning of α9 in Domain G2, as well as a Q_317_ residue in α12 of Domain CCL (Table 3). Interestingly, TLR2 SSGS_39–42_ segment and LcrV RKDS_53–56_ play a major role in forming this hydrogen bond network, and are involved in 8/13 hydrogen bonds formed (Fig. 6).

**Table 3:** Predicted hydrogen bonds between LcrV and TLR2 on the opposite side

LcrV residue	LcrV secondary structure	TLR2 residue	Bond length (Å)
R_53_	Loop between β1 and α2	S_40_	2.6
K_54_ (peptidyl carbonyl group)	Loop between β1 and α2	S_40_	2.6
K_54_ (peptidyl amino group)	Loop between β1 and α2	S_40_	4.0
K_54_	Loop between β1 and α2	G_41_	3.1
D_55_	Loop between β1 and α2	S_40_	4.1
S_56_	Loop between β1 and α2	S_27_	3.4
S_56_	Loop between β1 and α2	S_39_	3.9
S_56_ (side chain hydroxyl group)	Loop between β1 and α2	S_40_	2.6
S_56_ (peptidyl amino group)	Loop between β1 and α2	S_40_	3.2
E_57_	Loop between β1 and α2	S_29_	3.1
T_202_	Linker between α8 and α9	H_318_	3.2
E_205_	α9	Q_345_	3.8
Q_307_	α12	S_42_	3.9

### A structure-based mechanistic model for LcrV-TLR2 complex formation and the role of LcrV in immune response

**Fig. 7:**
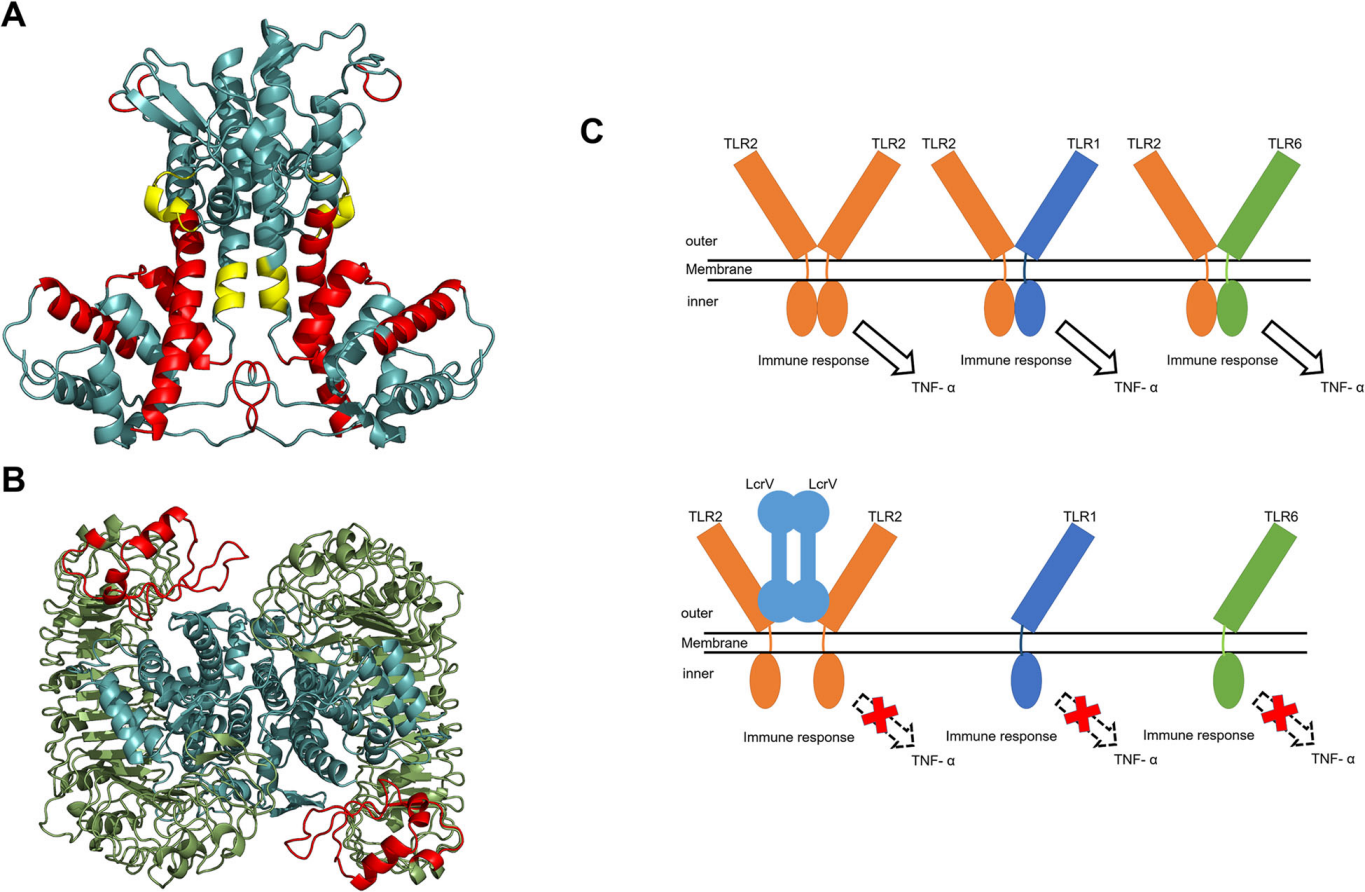
A proposed mechanistic model for LcrV function in immune repression. Panel (**a**), regions critical for LcrV-TLR2 complex formation, red color: regions critical for LcrV-TLR2 binding, yellow color: regions critical for LcrV-LcrV binding, the regions not critical to subunit binding are shown in blue; Panel (**b**), separation of TLR2 C-terminus by LcrV, blue color: LcrV subunits, green color: TLR2 subunits, red color: TLR2 C-terminus; Panel (**c**), proposed mechanistic model for LcrV in immune response repression, orange color: TLR2, blue color: TLR1, green color: TLR6, light blue color: LcrV, top: when not bound with LcrV, TLR subunits form dimers leading to immune response; bottom: when TLR2 dimers are bound with LcrV forming heterotetramers, TLR2 subunits are separated leading to the loss of immune response and TLR1/6 cannot form dimers with TLR2 which leads to the loss of immune response

From the modelled heterotetrameric structure of LcrV-TLR2 complex, a model for the role of each LcrV domain could be proposed (Fig. 7a). In the LcrV-TLR2 structure model, the formation of LcrV dimer is primarily due to the extensive interactions between CCL domains and the α8-containing loop region of Domain G2 (Y_201_-A_209_). The two TLR2 subunits are not directly associated in the structure model. Instead, they are held together via extensive interactions with both LcrV subunits. Several regions were found essential for the formation of the heterotetrameric complex in the structure model: the β strand containing loop (43–63) in Domain G1, α4 (92–107) in Domain G1, α6 and its linker to α5 (127–145) in Domain G1, the whole CCL domain, the loop region on the N-terminus of α12, and α8-containing loop region of Domain G2 (196–208).

One prominent phenomenon we observed in the LcrV-TLR2 complex structure model is that the LcrV subunits separate the two TLR2 subunits in the complex. In this configuration, the TIR-connecting C-terminus of TLR2 extracellular section were separated by two LcrV subunits, making it impossible for the formation of TIR dimers (Fig. 7b). Therefore, we propose that LcrV functions in inhibiting the immune response of white blood cells by inhibiting TIR dimer formation, the signal transduction via TLR2, and subsequent induction of inflammation factors such as TNF-α [25]. The formation of LcrV-TLR2 complex also competitively inhibits the binding of other toll-like receptors (such as TLR1 and TLR6) with TLR2 for immune response. A model of LcrV in immune response can be summarized in Fig. 7c.

## Discussion

A large body of literature discussed *Y. pestis* LcrV and its immunological repression function involving *H. sapiens* TLR2 and other proteins [1, 17–22, 24], yet the mechanistic insights on how LcrV binds to TLR2 for its function have been under controversy due to the lack of a LcrV-TLR2 complex structure. In this work, with a modelling-based approach, we successfully obtained a LcrV-TLR2 heterotetrameric complex structure model, from which a mechanistic model for the function of LcrV was proposed.

In this model, LcrV functions in spatially separating the two TLR2 subunits to prevent the formation of functional TIR dimers. LcrV may also recruit TLR2 and competitively prevent the formation of functional complexes of TLR2 and other TLR subunits. This model explains why D_203_-I_206_ and T_271_-S_300_ are so important in the function of LcrV [19, 20]: the former segment is the key to the binding of LcrV to LcrV, while the later segment is essential for the binding of LcrV and TLR2 [19]. The deletion of D_203_-I_206_ reduces the function of LcrV but cannot totally abolish it, as Domain G2 also helps the formation of LcrV dimer (Fig. 7a). However, the removal of T_271_-S_300_ not only removed the largest surface for LcrV-TLR2 interaction, but may also lead to significant change of Domain G1 structure, leading to the inability of LcrV to bind to TLR2, agreeing to previous findings [20].

The most striking feature of the structure model of LcrV-TLR2 is the extent of interactions involved in the maintenance of the structure. In addition to previously found key regions for function, as shown in Fig. 7a, all three domains of LcrV are involved in the binding between LcrV to LcrV, and LcrV to TLR2. These extensive interactions make the binding of LcrV to TLR2 resistance to mutation: minor mutations, even in critical binding regions, do not change the overall binding of LcrV and TLR2, and subsequently the effectiveness of LcrV. This feature makes it particularly difficult for host cells to resist LcrV, and *Y. pestis* invasion. Recent investigations showed the amino acid polymorphism in *Yersinia* LcrV proteins that enables immune escape [26, 27]. Interestingly, only one of the variable sites (E_205_) is involved in hydrogen bond formation, implicating the importance of this hydrogen bond network between LcrV monomers and between LcrV/TLR2 for its function.

In previous biochemical and immunological work, controversies stood on the mechanism of LcrV function: although research generally agreed that LcrV represses immunological factors such as TNF-α, whether this repression is mediated by stimulating IL-10 has been controversial [21, 22]. The mechanistic model established in this work supports the repression of TNF-α by LcrV as binding of LcrV with TLR2 prevents TIR dimers formation, therefore blocking TNF-α stimulation (Fig. 7c). The stimulation of IL-10, on the other hand, was not shown in this proposed model. Therefore, whether IL-10 stimulation is involved in the function of LcrV remains unknown, and further investigation is required to determine the role of IL-10.

Previous research showed large multimers of LcrV (> 200 kD) can stimulate TLR2 leading to IL-8 formation [21]. We suspect this stimulation is due to the formation of large LcrV_2n_-TLR2_2n_ aggregates which brings TLR2 moieties from different LcrV-TLR2 heterotetramers in close proximity, leading to immune response.

In addition to the regions proposed to be involved in LcrV-TLR2 complex formation, the role of the potentially active hairpin (P_220_-I_232_) structure in Domain G2 remains to be elucidated. Previous report showed that CD14 is involved in the interaction between LcrV and TLR2 [17]. We suspect that this region functions in binding to CD14 or other functional molecules for complete activity of LcrV-TLR2 complex.

## Conclusions

In conclusion, a structural model of the *Y. pestis* LcrV-*H. sapiens* TLR2 complex was constructed. The modelled structure is a LcrV_2_-TLR2_2_ heterotetramer. Analysis of the structure model revealed that the TLR2 subunits are held together by interactions between the two LcrV monomers and LcrV-TLR2 interactions. A mechanistic model was constructed from the modelled structure: The LcrV dimer separates the TLR2 subunits upon binding, leading to separation of the TIR domains linked at the C-terminus of TLR2 extracellular domain, thereby abolishing immune response; LcrV also binds to TLR2 and competitively prevents the formation of functional heterodimers of TLR2 and other TLRs. This model explains previous experimental phenomenon, and reveals more sites essential for the function of LcrV.

## Methods

### Modelling of protein structures and structure evaluation

The modelling of *Y. pestis* LcrV and *H. sapiens* TLR2 structures was performed using previously reported LcrV mutant structures (PDB ID: 4JBU, 1R6F) and *H. sapiens*-hagfish TLR2 fusion/*M. musculus* TLR2 protein structures **(**PDB ID: 2Z7X, 5D3I**)** as templates [1, 24, 25, 28, 29], and native *Y. pestis* LcrV/*H. sapiens* TLR2 sequences (Uniprot accession P0C7U7 and O60603). Modelling was performed using I-TASSER, SWISS-MODEL or Modeller [30–32]. Modelled structures were evaluated using ProQ, Verify3D, Procheck, Modfold, and QMEAN [33–37]. The best model was chosen for further optimization of the loop region using Modloop [38]. The final modelled structure is shown in Additional file 2.

### Modelling of LcrV-TLR2 complex structure

The structures of LcrV homodimer and LcrV-TLR2 heterodimers were modelled using GrammX [39]. The LcrV-TLR2 heterotetramer structure was constructed by manually matching LcrV in LcrV homodimers to LcrV-TLR2 heterodimers.

### Molecular dynamics simulation

Molecular dynamics simulation of the modelled LcrV-TLR2 structure in water environment was performed using the Nanoscale Molecular Dynamics program (NAMD) that was developed by the Theoretical and Computational Biophysics Group in the Beckman Institute for Advanced Science and Technology at the University of Illinois at Urbana-Champaign (http://www.ks.uiuc.edu/Research/namd/) [40].

### Protein structure visualization and measurement

Protein structure visualization and measurement of distances/dihedral angle was performed using the PyMOL Molecular Graphics System version 2.2.3.

## Supplementary information

**Additional file 1.** Quality evaluation of modelled structures.

**Additional file 2.** Modelled structure of LcrV-TLR2 heterotetrameric complex.

## Data Availability

The datasets used and/or analyzed during the current study are available from the corresponding author on reasonable request.

## Abbreviations

CCL: Coiled-coil linker
LcrV: Low Calcium Response V
NAMD: Nanoscale Molecular Dynamics program
T3SS: Type III Secretion System
TLR2: Toll-like Receptor 2
Yop: *Yersinia* outer membrane protein
