# Supplementary material for: Interactions between Yersinia pestis V-antigen (LcrV) and human Toll-like receptor 2 (TLR2) in a modelled protein complex and potential mechanistic insights
Source: BMC Immunol. 2019 Dec 16;20:48. doi: 10.1186/s12865-019-0329-5 (PMC6916237; doi:10.1186/s12865-019-0329-5)
Supplement: Supplementary file 1 — Additional file 1. Quality evaluation of modelled structures. [file 12865_2019_329_MOESM1_ESM.docx]

**Additional file 1. Quality evaluation of modelled structures.**

|  | ProQ_LG | ProQ_MS | Verify3D | Procheck | ModFold_p | QMEAN |
| --- | --- | --- | --- | --- | --- | --- |
| LcrV | 5.710 | 0.492 | 94.92% | 98.9% | 4.141×10^-4^ | 0.596 |
| TLR2 | 6.718 | 0.487 | 99.82% | 99.8% | 1.126×10^-4^ | 0.590 |
